# QT Interval Prolongation in Patients with Systemic Sclerosis—Are the Holter ECG Recordings a Better Option for QT Interval Evaluation?

**DOI:** 10.3390/medicina57030295

**Published:** 2021-03-22

**Authors:** Elena E. Saramet, Doina-Clementina Cojocaru, Sorin Ungurianu, Robert D. Negru, Codrina Ancuta

**Affiliations:** 1 Department of Internal Medicine, Gr T Popa University of Medicine and Pharmacy, 700115 Iasi, Romania; smaranda.saramet@gmail.com (E.E.S.); clementina.cojocaru@gmail.com (D.-C.C.); codrina_ancuta@yahoo.com (C.A.); 2 Department of Surgery, Dunarea de Jos University, 800008 Galati, Romania; sorinungurianu@yahoo.com

**Keywords:** systemic sclerosis, Holter ECG, QTc interval

## Abstract

*Background and Objectives:* Cardiac involvement in systemic sclerosis has important consequences on patient survival. Myocardial fibrosis and microcirculation involvement can generate arrhythmic complications, which can be associated with a higher death risk. QT interval prolongation is considered as a risk factor for ectopic ventricular events and can be evaluated using standard short ECG recordings or 24-h Holter ECG recordings. *Materials and Methods:* 39 patients with systemic sclerosis were submitted to a standard ECG recording at admission and 24-h Holter ECG monitoring. *Results:* QT interval values resulted from Holter ECG monitoring are higher than the values generated by the short-term ECG recordings. Holter ECG monitoring permits the detection of ventricular ectopy in patients with no events on standard ECG. *Conclusions:* In patients with systemic sclerosis, 24-h Holter ECG recordings can realize a more precise evaluation of the extent of QTc interval prolongation and ventricular ectopic events associated with myocardial involvement.

## 1. Introduction

Systemic sclerosis (SSc)—a disease that has a predominant prevalence in female patients [1], is characterized by an extensive fibrotic process of the conjunctive tissue, which interests multiple organs and by the presence of autoantibodies. SSc can be associated with cardiac, renal, gastrointestinal skin, and pulmonary (vascular or interstitial) involvement. The cardiac involvement is usually represented by myocardial fibrosis—some authors are considering that all the patients with systemic sclerosis are having a degree myocardial fibrosis [2], pericardial and coronary microcirculation involvement. Myocarditis is also a form a cardiac involvement found in SSc and is responsible for left ventricle dysfunction and thromboembolic events, suspected particularly in SSc patients with concomitant peripheral myopathy [3,4]. The myocarditis in SSc has a higher degree of myocardial fibrosis with near undetectable myocardial oedema, compared to those with other types of virus-negative myocarditis and the myocardial fibrosis proved to be directly correlated with the skin score and number of premature ventricular contractions (PVC) on a 24-h ECG Holter [5].

The prevalence of clinical conditions associated with cardiac involvement was estimated between 15–35% and are represented by pericardial effusions, myocardial ischemia, arrhythmias, and other ECG anomalies [6]. The most frequent ECG anomalies in systemic sclerosis patients are represented by *p* wave anomalies, non-specific anomalies of ST-segment and T wave, QRS microvoltage, and more subtle changes like the low values of the heart rate variability parameters, QT dispersion (QTd) increase, and prolongation of the corrected QT (QTc) interval [7,8]. Cardiac complications are the second cause of death in systemic sclerosis patients, and some studies are considering that sudden death is responsible for 13% of the deaths recorded in patients with systemic sclerosis [9]. Several studies confirmed that in patients with systemic sclerosis, the presence of ventricular arrhythmic events detected on standard short term ECG recordings or Holter ECG recordings is associated with an increased death risk [10,11] and the abnormal Holter results were correlated with the degree of myocardial fibrosis evaluated via delayed enhanced magnetic resonance imaging (DE-MRI) [12]. The QTc interval prolongation is a frequent ECG manifestation in patients with systemic sclerosis and is considered a major risk factor for ventricular arrhythmic events [13]. Our study aims to compare the rate of QTc interval prolongation identification using standard ECG recordings with the results generated by Holter ECG recordings, to compare the obtained values with reference values for QTc interval and to correlate these values with the arrhythmic events recorded during Holter monitoring to generate a more precise cardiovascular risk evaluation of the patients with systemic sclerosis.

## 2. Materials and Methods

The study included 39 patients with systemic sclerosis admitted to the II-nd Rheumatology Clinic of our hospital between January 2019 and December 2019. The inclusion criteria were represented by the diagnostic of systemic sclerosis according to ACR/EULAR 2013 classification, confirmed by clinical examination and laboratory tests. Patients with ischemic heart disease, arterial hypertension, cardiac failure, chronic pulmonary diseases, diabetes, atrial fibrillation, bundle branch block, and those under treatment with beta-blockers, antiarrhythmic drugs, or other drugs known for interfering with QT interval duration were excluded. The study protocol was approved by the Ethics committee of the hospital (approval code no 1/29.10.2015) and all patients signed an informed consent when included in the study.

On hospital admission, for all the patients, a standard short term ECG recording (10 s) was obtained. A Mindray BeneHeart12 electrocardiograph with automatic calculation of QT and QTc intervals durations was used. All the recordings were analyzed by one of the authors. All patients were submitted to a minimum of 24-h Holter ECG recording using 300-3A recorders from DM Software—USA with 10 electrodes cables and a sampling rate of 4096 Hz. All the recordings were revised and analyzed by one of the authors using Cardioscan 12 Premier software (DM Software USA). The software calculated the following parameters: QTc interval duration for all the recorded sinus beats, the mean value of QTc interval obtained for all the sinus beats recorded during Holter ECG monitoring, and maximal value of QTc and QT intervals recorded during Holter monitoring. For an individual patient, the software also calculated the percentage of sinus beats (reported to the total number of sinus beats recorded at the patient) having the absolute QTc interval duration included in 10 predefined ranges: <350 ms, 350–370 ms, 370–390 ms, 390–410 ms, 410–430 ms, 430–450 ms, 450–470 ms, 470–490 ms, 490–510 ms and >510 ms. Due to the fact that the number of female patients in our study was dominant, the reference QTc value for interval prolongation was set at 450 ms. The number of ventricular events (ventricular extrasystolic beats and ventricular tachycardia episodes) was recorded. No nicotine or coffee consumption was allowed during the 24-h monitoring.

Statistical analysis was realized using the Open STAT program. The data are presented as median and range or means ± standard deviation (SD). All variables were tested for normal distribution using the Shapiro-Wilks test. The t-Student and One-way ANOVA F parametric tests were used for all the parameters having a normal distribution. For non-normal distribution, the Mann-Whitney U test was used. Correlations were evaluated using Spearman or Pearson coefficients, as appropriate. Statistical significance was set at a *p*-value of 0.05 or less.

## 3. Results

Our study included 39 patients with systemic sclerosis, 29 patients being females. The mean age of the patients was 56.41 ± 11.26 years. Clinical characteristics of the patients are summarized in Table 1.

During the hospital admission, 30 patients were receiving IV treatment with prostacycline analogues (Ilomedin), 8 patients with methrotrexate, 4 patients with both prostacyclin analogues and methotrexate. Eight patients were receiving corticotherapy (mini-pulse therapy with 125 mg methylprednisolone intravenously for 3 consecutive days). During the 24-h Holter examination, none of the iv drugs indicated above were administered.

The mean value of the QTc interval duration obtained from standard short duration ECG recordings was 416.49 ± 29.28 ms. Using these types of recordings, there was no statistically significant difference (*p* = 0.31) between the value of QTc interval of the female patients compared to the QTc interval value obtained in male patients. Short term ECG recordings indicated a QTc interval > 450 ms in 7 patients (18%—4 males, 3 females). No arrhythmic events (ventricular or supraventricular) were identified in short term ECG recordings.

When 24-h recordings were used, the median value of the number of the sinus beats recorded at each patient for which the Holter analysis software had calculated the value of QT and QTc intervals was 100,911 beats (range 74,961–126,885 beats). The median value of the number of sinus beats recorded at each patient and having a QTc interval duration >450 ms was 53,054 beats (range 514–110,483 beats). The median value of the percentage of the sinus beats (reported to a total number of sinus beats analyzed for the same patient) having a QTc interval value higher than the cut-off value of 450 ms was 48.9% (range 0.5–99.65%). During the Holter ECG monitoring, all the patients presented sinus beats with QTc > 450 ms, while in 24 patients, QTc values higher than 500 ms were recorded.

The mean value of the QTc interval generated by the analysis of the sinus beats recorded on Holter ECG recordings was 447.05 ± 22.20 ms. The mean value of the QTc interval of the 29 female patients was 452.31 ± 19.10 ms—significantly higher when compared with male patients (431.80 ± 24.47 ms, *p* = 0.01). The ventricular ectopic (PVC) events identified on Holter ECG are presented in Table 2.

For all the patients, there was no significant correlation between the number of premature ventricular contractions recorded and the QT interval parameters analyzed from short and Holter ECG. But the number of the PVC recorded on Holter monitoring was significantly higher (*p* = 0.04) in patients with a diffuse form of SSc when compared with localized form.

When we separately analyzed the patients according to the form of the SSc (diffuse or limited), there were no statistic differences for the following ECG parameters analyzed: QTc from the standard ECG, QTc, max QTc, and QRS with QT > 450 ms from 24-h Holter monitoring. The comparative analysis of the patients according the presence of the corticoid treatment, did not show any statistical significant difference between the groups for the main ECG parameters analyzed (QTc from the standard ECG, QTc, max QTc, and QRS with QT > 450 ms from 24-h Holter monitoring) or the presence of PVC in 24-h recordings.

We were not able to identify any significant effect of methotrexate on analyzed ECG parameters or presence of PVC.

For all the patients, the usual blood biochemical parameters indicated in Table 3 were determined. In 17 patients, the Anti-Scl-70 antibodies were detected.

We also tested the correlations between biochemical parameters and the ECG parameters—significant results are presented in Table 4.

## 4. Discussion

Prolonged QT interval is considered an important predictor for arrhythmic events associated with potentially lethal complications. This was the reason many studies were conducted to evaluate QT interval duration in systemic sclerosis patients. Massie et al., on a multicenter cohort study, realized on 689 patients with systemic sclerosis, using short-term ECG recordings with automatic measurement of QT interval identified prolonged values of QTc interval (>440 ms) in 171 patients (25%) [14]. De Luca et al., using short-term recordings, found an 11% prevalence (11 cases) of patients with QTc duration >440 ms in a study that included 100 patients with systemic sclerosis [15].

Another study realized on 57 cases, using 10-min ECG recordings, identified 18 cases (31.5%) having a QTc interval value > 440 ms [13]. Other studies [13,16] confirmed that QTc values in patients with systemic sclerosis were significantly higher when compared with the values obtained from matched controls and were correlated with the severity of the disease evaluated by nail fold capillaroscopy or the presence of digital ulcerations. In our study, when short ECG recordings with automatic calculation of QTc were used, we identified seven cases (18%) with a QTc value > 450 ms. The percentage was concordant with the values obtained in other studies that set a reference value of 440 ms.

In our study, the number of cases did not change if the cut-off value was set to 440 ms. On Holter ECG recordings in all the patients, we detected sinus beats with QTc > 450 ms (in 24 patients we recorded QTc > 500 ms), suggesting that the patients with SSc were at risk of ventricular ectopic contractions even if the standard ECG was showing no QTc abnormalities. The median value of the number of sinus beats recorded at each patient and having a QTc interval duration > 450 ms was 53,054 beats (range 514–110,483 beats) and the mean value of QTc interval obtained by Holter ECG recordings (447.05 ± 22.20 ms) was significantly higher (*p* < 0.001) when compared with the mean value of the QTc interval automatically calculated using short ECG recordings (416.49 ± 29.28 ms), but the mean value of the QTc interval obtained using Holter recordings was not statistically significantly higher (*p* = 0.41) when compared with the cut-off value of 450 ms.

The association between serum autoantibody levels and QTc prolongation was still controversial and the results were conflicting. Similar to Massie et al. [14], we were not able to confirm an association between the presence of Anti-Scl-70 antibodies and QTc interval prolongation either using Holter ECG or standard ECG recordings. Other authors suggested a strong correlation between QT interval prolongation and the presence of Anti-Ro/SSA antibodies [17].

When we separately analyzed female vs. male patients, there was no significant difference (*p* = 0.31) of QTc interval value obtained using short term ECG recordings, a situation also confirmed by other studies [16,18], but when the Holter ECG recordings were used, the mean QTc interval value of female patients was significantly higher (*p* < 0.05) when compared to the value obtained in male patients, maybe related to the fact that the number of sinus beats having a QTc interval > 450 ms was significantly higher (*p* = 0.002) in female patients compared with male patients. The mean values of QTc interval obtained from female and male patients are not significantly higher when compared with reference values of 450 or 440 ms, respectively.

In our study we were not able to confirm any influence of the clinical form of the SSc on the ECG parameters analyzed. Also the corticosteroid treatment was not associated with any significant changes of the ECG parameters analyzed or increased number of PVC.

In our study, patients with diffuse forms of SSc recorded a significantly higher number of PVC compared to localized forms, which confirmed the findings of recent studies that the extent of skin involvement evaluated by the modified Rodnan skin score was directly correlated with the number of PVC recorded on a 24-h Holter [5].

The relationship between serum uric acid (SUA) and premature ventricular contractions and ventricular tachycardia was confirmed by several studies [19,20]. In our study, we were not able to identify a correlation between SUA and ventricular ectopy, but we confirmed a strong positive correlation between uric acid values and two electrocardiographic parameters extracted from Holter recordings: the mean value of QTc interval value and the number of QRS with QTc > 450 ms.

Our study had limitations due mainly to the relatively small number of patients included in the study, further studies including a higher number of patients with systemic sclerosis were necessary in order to validate our data.

## 5. Conclusions

In patients with SSc, the evaluation of the QT interval duration and arrhythmic events based only on the standard ECG recordings realized on hospital admission can underestimate the extent of the prolongation of the QTc interval. Recordings from a 24-h Holter ECG can realize a more precise evaluation of the extent of QTc interval prolongation and the presence of ventricular ectopic events associated with myocardial involvement.

## Figures and Tables

**Table 1:** Clinical characteristics of the patients included in the study.

Clinical Features	No of Patients
Limited cutaneous systemic sclerosis (SSc)	8 (20.5%)
Diffuse cutaneous SSc	31 (79.5%)
Raynaud phenomenon	14 (36%)
Arthritis	3 (7.7%)
Lung involvement	4 (10%)
Esophageal involvement	4 (10%)
Sicca Syndrome	1 (2.5%)
Skin ulcers	2 (5%)

**Table 2:** Ventricular ectopic events on Holter ECG.

ECG Holter Event	
PVC n/24 h (median-range)	50 (1–2447)
Patients with PVC n (%)	39 (100%)
Patients with PVC > 1000/24 h n (%)	2 (5%)
Patients with TV non sustained n (%)	3 (7.7%)
Patients with couplets n (%)	3 (7.7%)

**Table 3:** Mean values of the blood biochemical parameters.

Biochemical Parameter	Mean Value (±SD)
Uric acid (µmol/L)	231.97 ± 72.57
Total Cholesterol (mmol/L)	4.99 ± 0.90
Serum Creatinine (µmol/L)	83.98 ± 10.60
Alkaline phosphatase (U/L)	88.72 ± 23.90
Glucose (mmol/L)	5.64 ± 2.07
Gamma-glutamyltransferase (U/L)	27.25 ± 17.93
C-reactive Protein (CRP) (mg/L)	6.66 ± 3.4
Triglycerides (mmol/L)	1.38 ± 0.56
Blood urea nitrogen (mmoL/L)	12.45 ± 4.78

**Table 4:** Correlations between the QT interval and the biochemical parameters.

Biological Parameter	Mean QTc from Holter Recordings		QTc from Short Term ECG Recordings		Number of QRS with QTc > 450 ms on Holter Recordings	
	r *	*p*	r ^*^	*p*	r *	*p*
Uric acid	0.53	0.0005	0.32	0.043	0.54	0.0004
Glucose	0.05	NS	0.147	NS	−0.522	0.0006
Gamma-glutamyltransferase	0.23	NS	0.332	0.038	0.253	NS

* Pearson or Spearman correlation coefficient (as appropriate).

## Data Availability

The data presented in this study are available on request from the corresponding author. The data are not publicly available due to ethical reasons.
